# Mechanical Property Improvement in Dissimilar Friction Stir Welded Al5083/Al6061 Joints: Effects of Post-Weld Heat Treatment and Abnormal Grain Growth

**DOI:** 10.3390/ma15010288

**Published:** 2021-12-31

**Authors:** Amir Hossein Baghdadi, Zainuddin Sajuri, Azadeh Keshtgar, Nurulakmal Mohd Sharif, Armin Rajabi

**Affiliations:** 1 Department of Mechanical and Manufacturing Engineering, Faculty of Engineering and Built Environment, Universiti Kebangsaan Malaysia, UKM, Bangi 43600, Selangor, Malaysia; baghdadi.amirhossein@gmail.com; 2 School of Materials & Mineral Resources Engineering, Engineering Campus, Universiti Sains Malaysia, Nibong Tebal 14300, Pulau Pinang, Malaysia; srnurul@usm.my; 3 Center for Risk and Reliability, University of Maryland, College Park, MD 20742, USA; azadeh.keshtgar@gmail.com

**Keywords:** aluminum alloy, Al6061, Al5083, friction stir welding, heat treatment, abnormal grain growth

## Abstract

The 5083 and 6061(T6) aluminum (Al) alloys are widely used in transportation industries and the development of structural designs because of their high toughness and high corrosion resistance. Friction stir welding (FSW) was performed to produce the dissimilar welded joint of Al5083-Al 6061(T6) under different welding parameters. However, softening behavior occurred in the friction stir welded (FSWed) samples because of grain coarsening or the dissolution of precipitation-hardening phases in the welding zone. Consequently, this research intended to investigate the effect of the post-weld heat treatment (PWHT) method on the mechanical property improvement of the dissimilar FSWed Al5083-Al6061(T6) and governing abnormal grain growth (AGG) through different welding parameters. The results showed PWHT enhanced the mechanical properties of dissimilar joints of Al5083-Al6061(T6). AGG was obtained in the microstructure of PWHTed joints, but appropriate PWHT could recover the dissolved precipitation-hardening particle in the heat-affected zone of the as-welded joint. Further, the tensile strength of the dissimilar joint increased from 181 MPa in the as-welded joint to 270 MPa in the PWHTed joint, showing 93% welding efficacy.

## 1. Introduction

Aluminum (Al) alloys are remarkably adaptable materials that are commonly utilized in transportation industries and the development of structural designs [1,2]. Given their moderate strength, commendable toughness features, and high corrosion resistance, Al5083 and Al6061 are ideal for applications related to the automotive industry and fabrication of marine structures, pipelines, and aircraft components [3,4,5]. Magnesium and silicon are the main alloying elements in the heat-treatable alloy Al6061, whereas magnesium is the only main alloying element in the strain-hardened alloy Al5083 [6,7].

For the combined benefits of different characteristics of various aluminum alloys, dissimilar welding of Al alloys has received consideration and is increasingly being used in the design of lightweight structures in different industries [8]. Renowned as a versatile solid-state welding method, friction stir welding (FSW) is an alternative process to join Al alloys [9,10,11]. In FSW, material deformation occurs at a temperature lower than the melting point temperature of joined materials and consequently minimizes distortion and residual stresses in comparison with conventional welding methods [12,13]. Therefore, the issues that arise in the fusion joining of these alloys are absent in FSW [14]. However, a softening behavior or strength reduction also occurs in the welding zone of FSWed Al alloys but to a degree that is considerably lower compared with that in fusion welds [15]. Softening behavior results from the dissolution or coarsening of precipitation-hardened particles in the heat-affected zone (HAZ) [16,17]. The extent of strength reduction in the welding zone during FSW is dependent on the welding parameters applied during the welding process; however, strength reduction occurs despite the application of optimum weld parameters [18,19,20].

Post-welding processes can be performed on welded samples to improve the loss of strength in the welding of Al alloys by recovering precipitation-hardening particles and relieving residual stress or the average grain size reduction in the weld zone [21]. Hamed [22] carried out the natural aging process on the dissimilar FSWed Al7075-Al5083. The results showed no noticeable effect on the UTS of the FSWed specimens after 180 days of natural aging. Baghdadi et al. [23] performed T6-PWHT (solutionizing process at 535 °C for 1 h and artificial aging at 175 °C for 8 h) on the similar FSWed Al6061. PWHT diminished the Portevin–Le Chatelier (PLC) effect that occurred after the welding process and restored the mechanical properties of the welded samples to their original state due to the homogenous distribution of Mg_2_Si particles. Pabandi et al. [24] investigated PWHT on the dissimilar FSWed Al6061-Al2024. A welding efficiency of 80% was achieved in the PWHTed samples with the emergence of AGG due to solution heat treatments performed at 520 °C. Paidar et al. [25] studied the effect of PWHT (solution heat treatment at 520 °C for 1 h and artificial aging at 165 °C for 18 h) on dissimilar FSWed Al2024-Al6061. A minor improvement of 13 MPa was obtained in the shear strength of PWHTed samples under optimum welding parameters due to the recovery of the strengthened phases of Al_2_CuMg and MgZn_2_. Furthermore, Zhang et al. [26] reported the effect of PWHT on dissimilar FSWed Al2024-Al7075. The results showed that AGG occurred in the SZ, and the UTS of PWHTed samples was not improved by PWHT due to the lack of mixing of materials in the weld zone. 

To date, studies of the effect of heat treatment after the dissimilar FSW of Al5083 and Al6061(T6) are lacking. Therefore, the effect of PWHT on the microstructure and mechanical properties of dissimilar FSWed Al5083-Al6061(T6) samples must be comprehensively studied. Accordingly, this research intended to examine the effect of PWHT on the microstructure, mechanical properties, and serration-flow behavior of the stress–strain curves of dissimilar FSWed samples under different welding parameters.

## 2. Materials and Methods

Commercial wrought aluminum alloys of Al6061(T6) and Al5083 were used as base materials (BMs) in this research. Table 1 presents the composition of these alloys and their mechanical properties. The sample size of 150 × 50 × 4 mm^3^ was considered for the dissimilar welding process of Al6061(T6) and Al5083.

**Figure 1:**
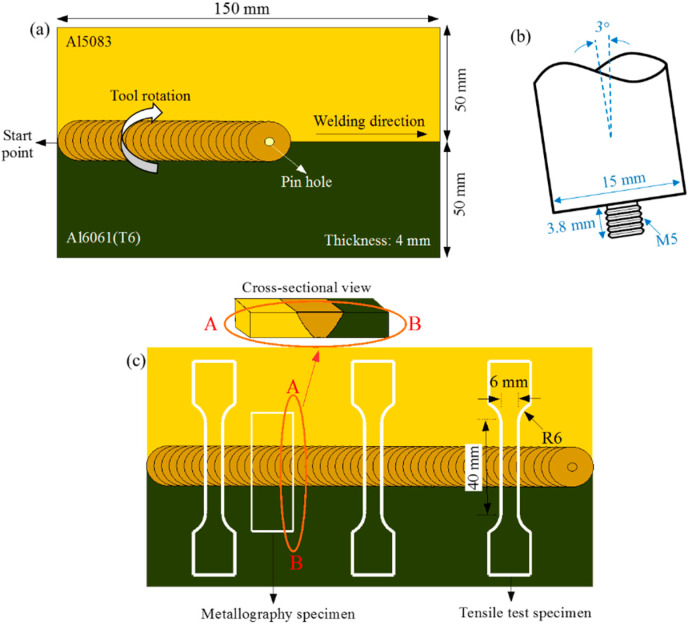
Schematic of the FSW process: (**a**) configuration of the welding joint, (**b**) welding tool, and (**c**) the orientation of samples cutting for microstructure and mechanical testing.

Before the welding process, the oxide layers on the surfaces of materials were removed using a steel brush and then washed with acetone. The butt joint configuration was considered in the dissimilar FSW process as shown in Figure 1a. During the FSW process, Al5083 was located at the advancing side (AS), and Al6061(T6) was positioned at the retreating side (RS). The H13 tool steel with a length of 3.8 mm and a shoulder diameter of 20 mm was used as the FSW tool in the welding process. The FSW tool was tilted by three degrees during the welding process. Table 2 shows the welding parameters and their combinations applied in the welding and PWHT processes. The tool rotational speed varied from 800 rpm to 1400 rpm, and traveling speeds were set to 100 and 400 mm/min. Two sets of joints, i.e., as-welded (FSW) and PWHT samples, were produced to investigate the effect of PWHT on the dissimilar welded joint properties. The PWHT process was carried out as follows: solutionizing at 535 °C for 1 h, quenching in water, and artificial aging at 175 °C for 8 h.

Figure 1c shows the location of the cross-section that was used to observe the microstructure of dissimilar FSWed samples under both conditions. Before the microstructural observation, cold-mounted samples were manually ground by emery papers ranging from #800 to #1800. Then, a diamond polishing suspension with a particle size of 1 µm was used to polish the samples. The microstructure of the welded samples was revealed via the electrochemical etching method using Barker’s reagent at 20 VDC under an optical microscope. The tensile test was performed on a 100 kN-capacity Zwick/Roell tensile test machine at room temperature and a strain rate of 10^−3^ s^−1^ following the ASTM standard E8M-04.

Figure 1c shows the schematic of the tensile test sample. The tensile strength was calculated based on the average of three stress values, and joint efficiency was obtained by dividing the ultimate stress of the welded joint by that of the Al6061(T6)-BM and then multiplying it by 100 $\left[W_{eff}=\left(\sigma_{UTS\;\left(Welded\right)}/\sigma_{UTS\;(Al6061\left(T6\right)}\right)\times 100\%\right].$ A micro-hardness test was performed on the cross-section of dissimilar FSWed samples using A Zwick Vickers hardness (HV) tester with a load of 0.1 kgf and a dwell time of 15 s per the ASTM standard E384. In addition, field emission scanning electron microscopy (SEM) was used to investigate the fracture modes of the samples after the tensile test.

## 3. Results and Discussion

### 3.1. Microstructure

After the welding process, the weld surfaces of the samples were visually inspected to check the welding defects in the welded samples. No welding defects, such as tearing, crack, and porosity, were observed on the weld surfaces. In addition, the weld appearance had smooth semicircular traces with a flash pushed out from the welding zone due to the welding process. 

**Figure 2:**
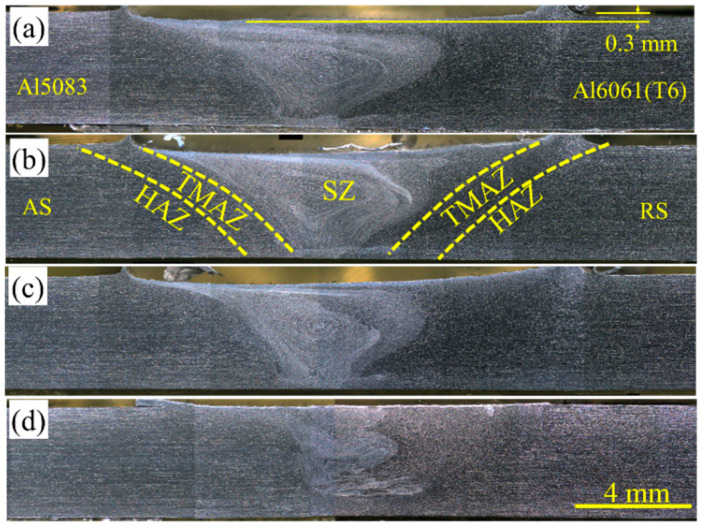
Macro photos of the dissimilar joint of Al5083-Al6061(T6) under different welding parameters; Samples (**a**) A-1, (**b**) C-1, (**c**) D-1, and (**d**) C-4.

Figure 2 shows the cross-sectional macro photos of the dissimilar joint of Al5083-Al6061(T6) under different welding parameters. The macrographs presented no common welding defects, such as crack, porosity, or cavities. Notably, Al5083 was placed in AS, whereas Al601(T6) was located in RS. Three welding zones, namely, SZ, TMAZ, and HAZ, were obtained. The pin penetration was 0.3 mm, and it remained constant under all the welding conditions (Figure 2). SZ in the dissimilar FSW was evident, and the complex structure of Al5083 and Al6061(T6) was demonstrated in the SZ with a flowing shape mostly composed of an AS material (Al5083) compared with that in a similar FSWed joint [27,28]. 

According to the obtained results, a clear borderline was found between SZ and TMAZ at the Al5083 side, whereas no distinct border was observed at the Al6061(T6) side largely because of the reaction of materials with the etching reagent. In other words, the material transported around the tool pin and deposited on the weld line at the RS was notably less than that at AS. The matter was likely formed mostly due to sufficient heat input and stirring impact enhancement, indicating that adequate mixing occurred on AS rather than on RS [22,24,29]. Furthermore, the comparison showed that the mixing of materials at SZ was increased by the increased rotational speed from 800 rpm to 1400 rpm. This finding may be attributed to the high heat generation and the peak temperature of the samples [22]. In addition, the amount of the dragged material and the mixing of the two BMs in the SZ were decreased by increasing the welding travel speed from 100 mm/min to 400 mm/min.

**Figure 3:**
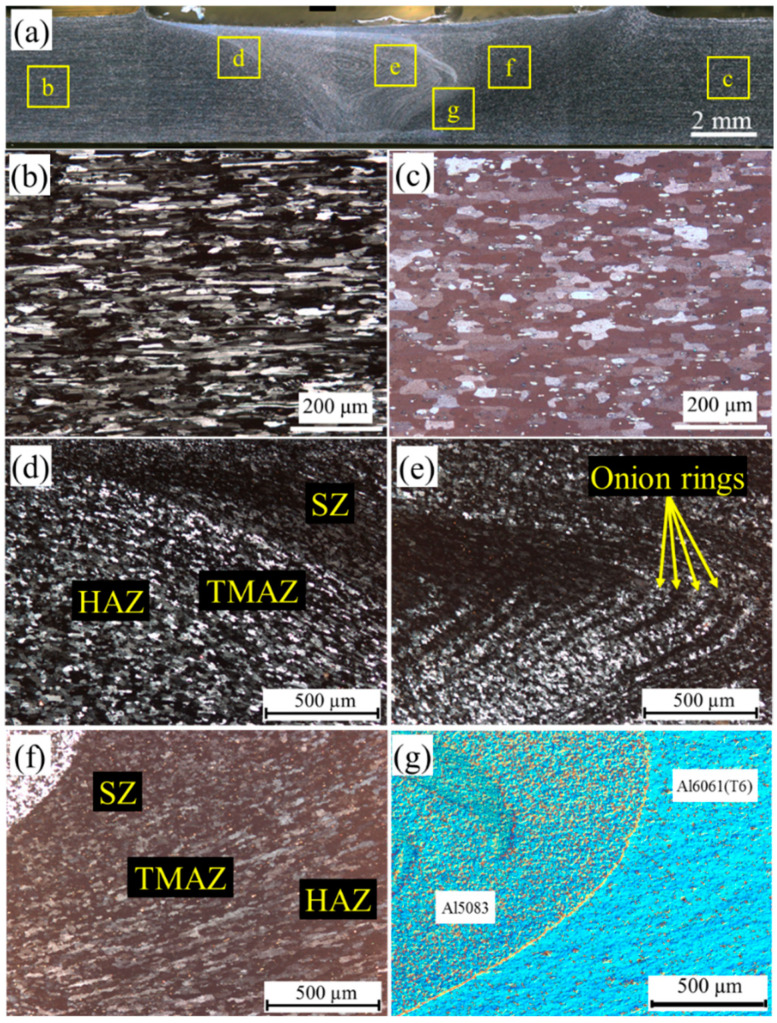
Microstructure of the dissimilar joint of Al5083-Al6061(T6) at Sample C-1; (**a**) cross-sectional view, (**b**) Al5083-BM, (**c**) Al6061(T6)-BM, (**d**) AS, (**e**) SZ, (**f**) RS, and (**g**) differential interference contrast (DIC) image of SZ.

Figure 3 presents the influence of the FSW process on the welding zone microstructure of welded sample at welding parameters of 1200 rpm and 100 mm/min. Figure 3a shows the cross-sectional view of the welding zone. The shape of SZ was asymmetric, and SZ was limited by a thin layer of BMs. The Al5083-BM sheet revealed a typical-rolled microstructure of the inhomogeneous grains aligned parallel to RD as presented in Figure 3b. Moreover, an elongated microstructure was observed in the Al6061(T6)-BM microstructure (Figure 3c). The average grain sizes of 29 and 36 µm were obtained for Al5083-BM and Al6061(T6)-BM, respectively. 

Figure 3d,f show the high-magnification images of the region (d) at the Al5083 side and region (f) at the Al6061(T6) side, respectively. The welding thermal cycle occurred at the HAZ, and no plastic deformation transpired in this zone during the welding process. Consequently, the HAZ showed a microstructure similar to the base metal with a large average grain because of the welding heat input [30,31]. Figure 3d,e illustrate the TMAZ microstructure where the main grains were ringed and stretched in an upward flowing pattern around the nugget. The partial dynamic recrystallization was evaluated at a high strain rate and temperature given that the grains in this zone were strongly deformed.

The SZ revealed a fine grain microstructure because of the mixing of materials directly by the FSW tool. In addition, the SZ exhibited dynamic recrystallization under high temperatures, intense plastic deformation, and a high strain rate applied to materials [32]. Onion ring patterns were detected in the SZ (Figure 3e). Onion rings are described as properly mixed materials in the welding zone during the FSW. The light color was believed to be Al5083, whereas the dark one was Al6061(T6).

Onion rings occur as a result of the ejection of cylindrical sheets of materials in each revolution as they move forward. Given that the influence of tool revolution on materials diminishes in the region away from the weld center, the thickness of bands in an onion ring was large in the center of SZ and thin in the direction of the BMs. Figure 3g presents the DIC image of SZ and TMAZ at the RS of the dissimilar joint sample. The border of SZ and TMAZ was evident in RS. This figure distinguishes Al5083 in the mixed microstructure of the SZ and Al6061(T6) in TMAZ at the RS. 

**Figure 4:**
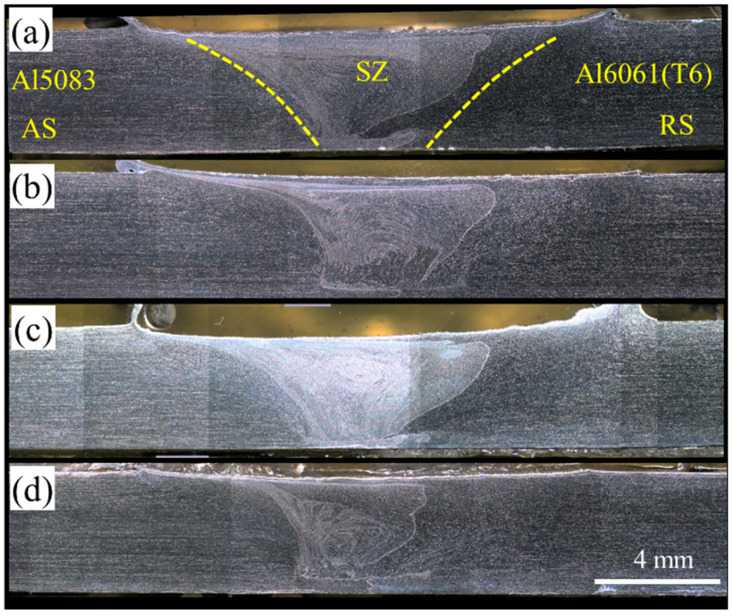
Macro photos of PWHTed Al5083-Al6061(T6) Samples; (**a**) A-1(PWHT), (**b**) C-1(PWHT), (**c**) D-1(PWHT), and (**d**) C-4(PWHT).

After FSW of the dissimilar joint of Al5083-Al6061(T6), the T6 heat treatment included solutionizing and precipitation hardening on the dissimilar FSWed Al5083-Al6061(T6) as PWHT to evaluate its effect on the microstructure and mechanical properties of the joints, which were a complex microstructure of heat-treatable and non-heat-treatable Al alloys. Figure 4 shows the cross-section of PWHTed Al5083-Al6061(T6) under different welding parameters. The welding zone was evident after a PWHT similar to the as-welded condition. Grain growth is a common phenomenon occurring in the welding zone after PWHT. Thus, the microstructure of the PWHTed samples in the welding zones was investigated to evaluate the effects of PWHT under different welding parameters.

**Figure 5:**
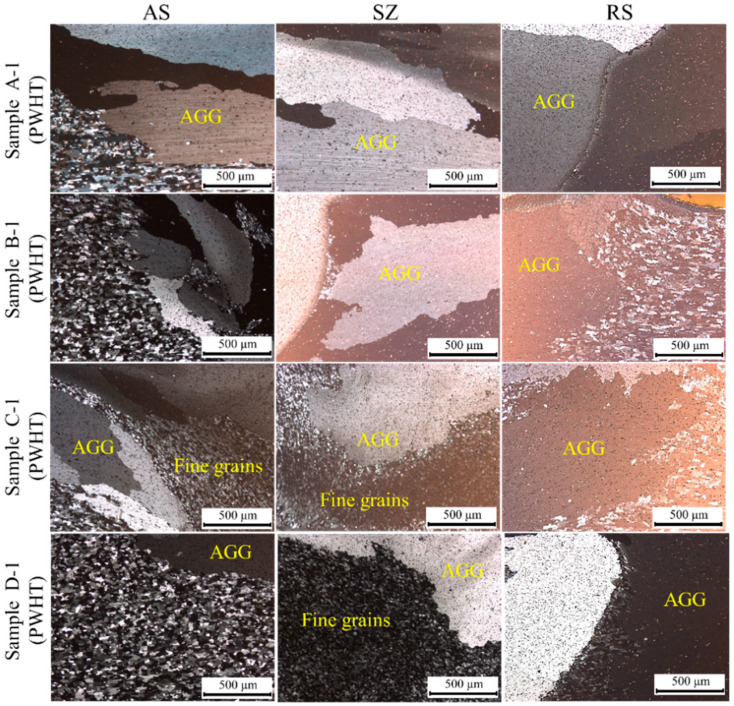
AGG occurs in the microstructure of PWHTed Al5083-l6061(T6) at a constant travel speed of 100 mm/min.

The main effect of PWHT was grain growth in the welding zones. Figure 5 shows the AS, SZ, and RS of the welding zones of the PWHTed samples under different welding parameters. The TMAZ on both sides of the welding zone, i.e., AS and RS, was not found in the microstructure of the PWHTed samples compared with that under the as-weld condition. The grain size in the SZ increased to several hundred micrometers and millimeters as observed in Figure 5. 

This phenomenon of microstructure instability occurring in the SZ is called AGG. AGG generally occurs when normal grain growth is suppressed [33]. In general, three factors influence the AGG in microstructure: (i) texture effect, i.e., anisotropy in grain boundary energy and mobility; (ii) decrease in pinning effect due to particle dissolution; (iii) nonuniform grain size distribution [34,35]. Thus, the balance between the thermodynamic driving force inducing AGG and the pinning force or barrier migration of grain boundaries is a critical feature that should be considered.

The microstructure of SZ was recrystallized and dominated by high-angle grain boundaries due to the random texture distribution in the FSW zone [36]. Variation in energy and mobility between the high-angle grain boundaries did not significantly restart recrystallization when a static high temperature was applied. Furthermore, Mn and Fe, the main elements of the second-phase particles in Al5083, were difficult to dissolve in the Al matrix because the dissolution temperature of these second phases is high; the Mg_2_Si intermetallic compound, which is thermally stable, existed in the SZ [37,38]. Thus, these second-phase particles remained unchanged during FSW at different rotational or travel speeds and PWHT at a high temperature (less than 550 °C) [39]. 

PWHT applied to the dissimilar FSWed samples was carried out under the same condition. Therefore, we should focus on the influence of welding parameters, which showed a significant effect on obtaining different microstructures in the welding zones due to the variation in heat input and plastic deformation and the presence of different material flows. The SZ was fully covered by AGG in Samples A-1(PWHT) and B-1(PWHT). However, less than a quarter of the thickness of Samples C-1(PWHT) and D-1(PWHT) experienced AGG, and the remaining parts of the welding zone revealed fine grains after PWHT.

AGG was also observed in the PWHTed sample at a constant rotational speed of 1200 rpm, i.e., Samples C-1(PWHT), C-2(PWHT), C-3(PWHT), and C-4(PWHT). The AGG was increased by an increase in the travel speed from 100 mm/min in Sample C-1(PWHT) to 400 mm/min in Sample C-4(PWHT). The entire thickness of the SZ microstructure underwent AGG in Samples C-3(PWHT) and C-4(PWHT). Moreover, two-thirds of the PWHTed sample exhibited AGG in Sample C-2(PWHT). 

Welding parameters influenced the heat input and corresponding thermal cycle. Heat input is inversely related to the weld pitch (W/P). A low W/P corresponds to a high heat input; consequently, AGG occurs to a low extent [40]. In our study, W/P at a welding parameter of 1400 rpm was smaller than that of other FSWed samples (Table 3). The SZ of the PWHTed sample exhibited a low extent of AGG. Moreover, the W/P increased as the travel speed increased at constant rotational speed. Consequently, the SZ of the PWHTed sample slightly experienced AGG at a constant rotational speed of 1200 rpm and travel speed of 100 mm/min. Therefore, AGG can be controlled by welding parameters, and a good agreement was observed between the revealed microstructure and heat input in the PWHTed Al5083-Al6061(T6) samples.

### 3.2. Mechanical Properties 

#### 3.2.1. Tensile Properties

After the FSW process, a tensile test was performed on the dissimilar joint at the direction normal to the welding direction. Table 4 lists the results of tensile strength of dissimilar joint samples, including UTS, elongation, and joint efficiency, under different welding conditions. In Table 4, the tensile strength was obtained from the average of three stress values, and the joint efficiency was calculated based on the ratio of the UTS of the welded joint to the lower tensile strength of the base materials, i.e., the ultimate stress of Al6061(T6)-BM, multiplied by 100.

The tensile strength and elongation of the dissimilar joint samples were lower than those of BMs. The tensile results showed a reduction of 39% in FSWed samples compared with the BM due to grain coarsening in the HAZ and over-aging of the strengthening phases in Al6061(T6)-BM. Grain coarsening in the microstructure was the main reason for the strength reduction in FSWed samples and failure at the HAZ of the joint. Different rotational speeds did not significantly affect the tensile strength at a constant travel speed of 100 mm/min. The highest tensile strength of 181 MPa was obtained in Sample C-1 at a constant travel speed of 100 mm/min. Zhou et al. [41] reported the highest tensile strength at 1200 rpm amongst the tested rotational speeds because of the existence of a good homogeneous microstructure at a high rotational speed.

However, the UTS of the joint increased by about 14% by increasing the travel speed from 100 mm/min to 400 mm/min at a constant rotational speed of 1200 rpm. The reason is that less heat was absorbed by the welding zone, leading to the decrease in the average grain size in HAZ due to the increase of travel speed at a constant rotational speed during welding. Saeidi et al. [42] reported that the tensile strength of the dissimilar joint of Al5083 increased as the travel speed increased at constant rotational speeds. As the tensile strength increased, the welding efficiency increased and reached 71% in Sample C-4. Furthermore, an increase in the travel speed did not significantly affect elongation, which was approximately 5.7% under all conditions. This decrease in ductility after welding can be attributed to the limited plasticity (i.e., strain concentration) in the joint samples subjected to the tensile test.

**Figure 6:**
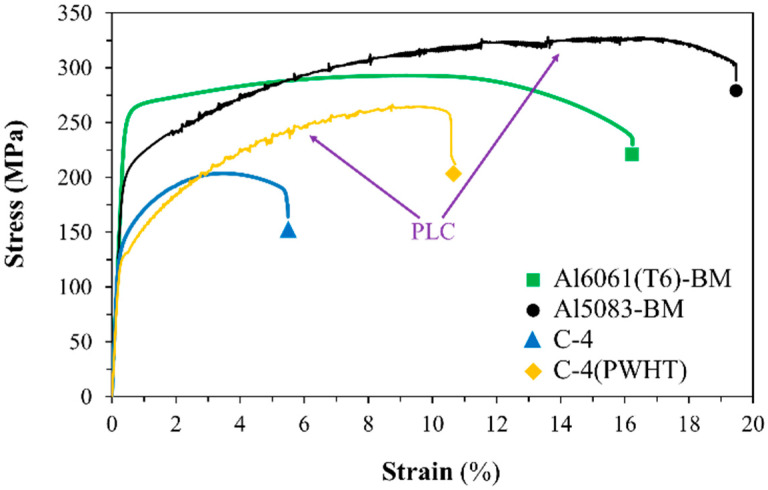
Tensile properties of FSWed and PWHTed of the Al5083-Al6061(T6) dissimilar joint.

Figure 6 presents the stress–strain curves of the dissimilar FSWed, PWHTed, Al5083-BM, and Al6061(T6) samples. The PLC effect was observed as a serration behavior in the dissimilar joints before *σ_UTS_*, as pointed by the arrow in Figure 6, due to plastic instability during the tensile test [23,43]. The PLC effect is due to transient interactions between mobile clouds of impurities (diffusing solute atoms) and glide dislocations [44]. An Mg atom containing Al5083 is larger than an Al atom in the Al matrix, thereby hindering the dislocation movement and increasing the strength of an Al alloy [45]. As a result, the PLC effect is linked to pinning and unpinning, whereas a pinned process occurs when barriers made from forest dislocations and grain boundaries prevent the movement of dislocation. Conversely, an unpinned process and a decrease in stress during the tensile test occur when dislocation can be overcome from the solute cloud [46]. Figure 6 shows the stress–strain curve of Sample C-4(PWHT) as a function of PWHT at a rotational speed of 1400 rpm. 

The PWHT improved the mechanical properties and increased the ductility of the dissimilar joint of Al5083-Al60661(T6) samples. However, the microstructure of the PWHTed samples had an elongated grain and a prolonged AGG in the welding zones. These observations were different from the mechanical properties obtained after PWHT. According to the well-known Hall–Petch equation, the tensile strength should decrease as the grain size increases. Recent studies reported that the effect of second-phase particle distribution on the mechanical properties of the Al5083 FSWed joint should be considered rather than grain size [24,41]. Thus, an increase in the tensile properties of the PWHTed samples can be attributed to the formation of precipitation-hardening particles, especially in Al6061(T6), which were recovered after PWHT. 

Table 4 shows the effect of PWHT on the tensile properties of the dissimilar joint of Al5083-Al6061(T6) compared with that of the dissimilar FSWed joint. The maximum ultimate stress of 235 MPa was achieved with an elongation of 7.1% in Sample C-1(PWHT). Thus, the welding efficiency changed from 62% under the as-welded condition to 81% for Sample C-1(PWHT). Further, the tensile properties increased as the travel speed increased from 100 mm/min to 400 mm/min possibly because of the good mixing of Al5083 and Al6061(T6) at a low heat input and the precipitation hardening effect after PWHT in the SZ. In addition, the yield stress of 163 MPa and maximum tensile strength of 270 MPa were achieved in Sample C-2(PWHT). 

From the results presented, PWHT is a beneficial post-welding process that can recover and improve the mechanical properties of FSWed heat-treatable Al6061 alloys. However, as discussed in the previous section, the critical problem of PWHT is grain growth or AGG in the microstructure of the PWHTed sample. AGG can affect the mechanical and fatigue properties of the FSWed samples for engineering structure applications. However, in this study, by controlling the welding parameters, i.e., the rotational speed at 1200 rpm and the travel speed at 200 mm/min, the AGG effect was suppressed and cancelled out by precipitation hardening from the T6 heat treatment, which resulted in the increased ultimate strength of the joint from 181 MPa to 270 MPa at 93% welding efficiency.

#### 3.2.2. Fractography

**Figure 7:**
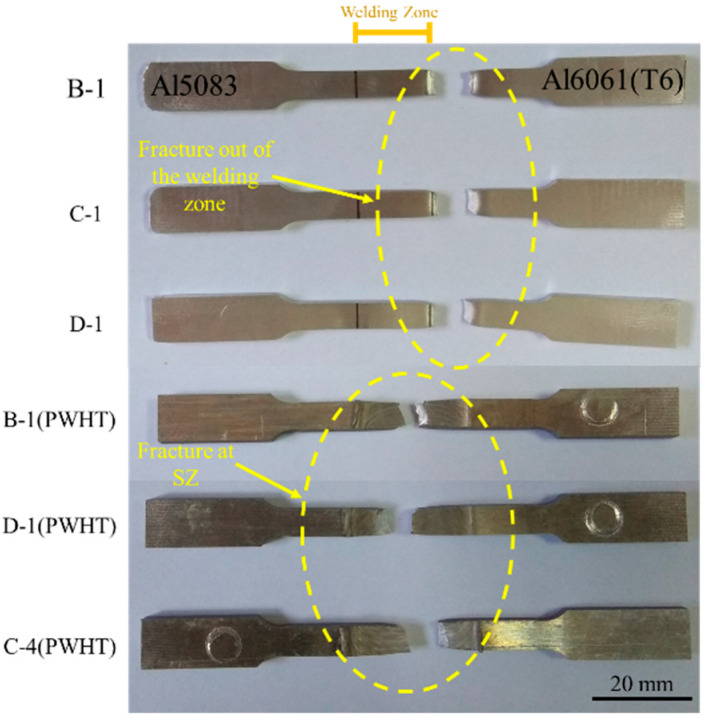
Fracture location of the dissimilar joint of Al5083-Al6061(T6) after the tensile test.

Figure 7 presents the fracture location of the dissimilar FSWed samples under different welding parameters. Fracture and necking occurred at the HAZ of Al6061(T6) in all the dissimilar joint samples. The overview of fracture location confirmed that it was outside the welding zone (Figure 7). The PWHTed samples exhibited the necking phenomenon at the fracture location. Notably, fracture and necking occurred at the interface of Al5083 and Al6061(T6) in the SZ. This finding showed that the fracture location moved from the HAZ in the as-welded samples to SZ in the PWHTed samples due to PWHT. The stress–strain curve behavior was attributed to the moving failure position (Figure 6). 

The mechanism of the serration flow behavior of dissimilar joints can be clarified and specified after the tensile test by checking the failure position in the dissimilar joint, which occurred in the HAZ at the RS and SZ in FSWed and PWHTed samples, respectively. However, the stress–strain curve behavior of the PWHTed Al5083-Al6061(T6) samples was different from that under the as-weld condition in terms of serration flow in the plastic region. The serration flow behavior of the PWHTed Al5083-Al6061(T6) samples was similar to that of Al5083-BM, which presented a severe serration flow behavior on the stress–strain curve due to the shifting of fracture location from RS to SZ toward the Al5083 side in PWHTed samples.

### 3.3. Hardness Profile 

**Figure 8:**
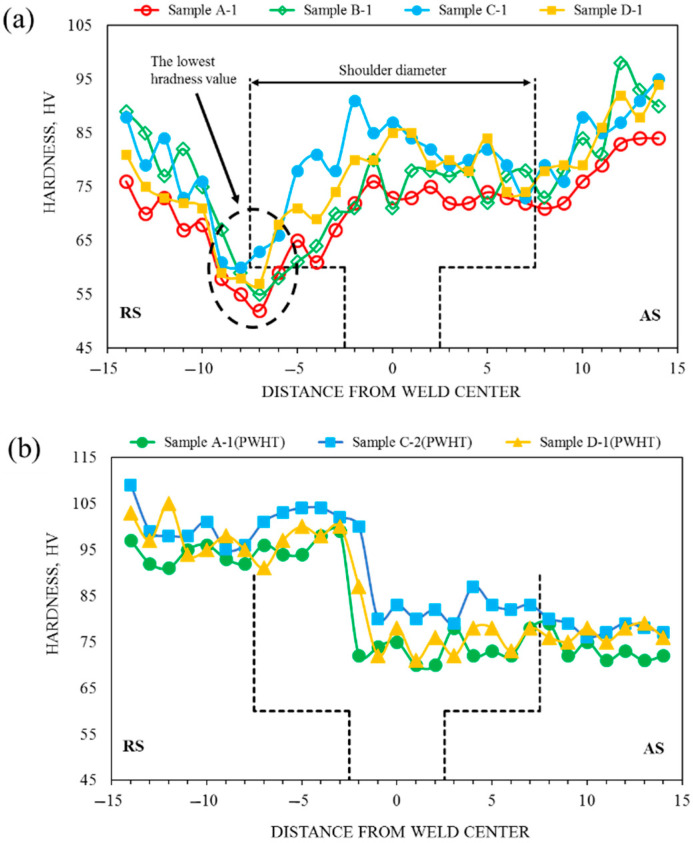
Hardness profile of the dissimilar joint of Al5083-Al6061(T6); (**a**) as-welded and (**b**) PWHTed conditions. The black dotted line is the tool pin profile.

The hardness (HV) distribution profile was determined to evaluate the hardness properties of the dissimilar joint of Al5083-Al6061(T6) samples under different welding parameters (Figure 8). The hardness profile of the dissimilar joint samples had the same behavior under as-welded conditions. In addition, a fluctuating hardness distribution profile was obtained from the dissimilar joint samples corresponding to three distinct welding zones, namely, SZ, TMAZ, and HAZ. 

The lowest HVs were detected at the HAZ adjacent to TMAZ on the RS, in which Al6061(T6) was placed on that side under all welding conditions. The lowest hardness values of 52, 55, 60, and 57 HV (Figure 8a) were obtained at rotational speeds of 800, 1000, 1200, and 1400 rpm, respectively. The measured HVs of Al6061(T6)-BM and Al5083-BM ranged from 93 to 97 and from 92 to 95, respectively. In general, the hardness properties in the welding zone of the dissimilar joint samples decreased compared with those of BMs, i.e., Al5083-BM and Al6061(T6)-BM, under different welding parameters. The plate underwent coarsening and solutionizing of the strengthening elements during welding. In addition, HAZ experienced the over-aging caused by the coarsening of strengthening elements due to heat input during the joining process. Thus, these cases caused a reduction in the hardness in the welding zones, especially in RS, which was heat-treatable Al6061(T6). This result is consistent with the failed dissimilar joint samples after the tensile tests, in which the fracture location was placed in HAZ due to a low hardness value.

In PWHTed samples, the hardness profile was obtained with the same behavior, showing that the hardness properties were improved by PWHT (Figure 8b). Notably, the improvement of hardness properties was significant in the RS, which was Al6061(T6). The hardness properties of Al6061(T6) were enhanced because of the homogeneous distribution of precipitation hardening after PWHT [47]. However, the hardness properties of Al5083 decreased in AS because of the coarsening of the hard particle and grain growth in BM. With AGG in the welding zone after PWHT, the hardness test of the PWHTed samples showed that precipitation hardening was the main factor that increased the strength of A6061(T6) rather than the average grain size. Thus, a good agreement was observed between the results of the tensile and hardness tests. The hardness profile of the PWHTed samples showed the same properties with low fluctuation on BMs, and the lowest hardness value was located at the interface of the PWHTed samples. The fracture location of the PWHTed samples occurred at the interface of the SZ. This finding was consistent with the results obtained via the hardness test.

## 4. Conclusions

This study was performed to investigate the effect of PWHT and FSW parameters on microstructure and mechanical properties of dissimilar joints of Al5083-Al6061(T6). The conclusions obtained from the investigation are summarized below.

1. Sound joints were obtained with no welding defects, such as cracks, porosity, and tunnel, under various welding parameters. The highest joint efficiency was obtained from Sample C-4 (1200 rpm and 400 mm/min) with a value of 71%. A decrease in the weld strength was observed in the as-welded samples due to the dissolution of precipitation hardening particles in the HAZ at RS, which was Al6061(T6).
2. By controlling the welding parameters, i.e., the rotational speed at 1200 rpm and the travel speed at 200 mm/min (Sample C-2 (PWHT)), the AGG effect was suppressed and cancelled out by the homogeneous distribution of precipitation hardening phases in the welding zone from the appropriate T6 heat treatment, which resulted in the increased ultimate strength of the joint from 181 MPa in the as-welded joint to 270 MPa in PWHTed joint at 93% welding efficiency.
3. The hardness properties were improved from 55 HV in the as-welded joint to 95 HV in PWHTed joint due to homogeneous distribution of precipitation hardening phases in the welding zone after PWHT, which caused a shifting of the fracture location from HAZ at RS in the as-welded joint to SZ of PWHTed joint after the tensile test.

## Figures and Tables

**Table 1:** Chemical compositions and tensile properties of the BMs.

Material	Chemical Composition (wt. %)							Yield Stress (MPa)	Tensile Stress (MPa)
	Al	Mg	Si	Mn	Fe	Cu	Zn		
Al6061(T6)	Balance	0.80	0.40	0.03	0.70	0.39	0.16	255	290
Al5083-H111	Balance	4.46	0.25	1.00	0.45	-	0.03	202	325

**Table 2:** Sample name and welding parameters of dissimilar joints of Al6061(T6)-Al5083.

Welding Process			PWHT Process
Sample Name	Rotational Speed (rpm)	Travel Speed (mm/min)	Sample Name
Sample A-1	800	100	Sample A-1(PWHT)
Sample B-1	1000	100	Sample B-1(PWHT)
Sample C-1	1200	100	Sample C-1(PWHT)
Sample C-2	1200	200	Sample C-2(PWHT)
Sample C-3	1200	300	Sample C-3(PWHT)
Sample C-4	1200	400	Sample C-4(PWHT)
Sample D-1	1400	100	Sample D-1(PWHT)

**Table 3:** Weld pitch under different welding parameters.

Sample	Welding Rotational Speed (rpm)	Welding Travel Speed (mm/min)	W/P
Sample A-1(PWHT)	800	100	1/8
Sample B-1(PWHT)	1000	100	1/10
Sample C-1(PWHT)	1200	100	1/12
Sample C-2(PWHT)	1200	200	1/6
Sample C-3(PWHT)	1200	300	1/4
Sample C-4(PWHT)	1200	400	1/3
Sample D-1(PWHT)	1400	100	1/14

**Table 4:** Tensile properties of dissimilar FSWed Al5083-Al6061(T6).

Sample	Ultimate Tensile Strength (MPa)	Joint Efficiency (%)	Sample	Ultimate Tensile Strength (MPa)	Joint Efficiency (%)
A-1	173	60	A-1(PWHT)	231	80
B-1	177	61	B-1(PWHT)	232	80
C-1	181	62	C-1(PWHT)	235	81
C-2	193	67	C-2(PWHT)	270	93
C-3	202	70	C-3(PWHT)	267	92
C-4	207	71	C-4(PWHT)	265	91
D-1	176	61	D-1(PWHT)	229	79
